# Mitochondrial genome of a Bolivian River Dolphin (*Inia boliviensis*)

**DOI:** 10.1080/23802359.2025.2544682

**Published:** 2025-08-11

**Authors:** Kristin Coury, Ellen Bronson, Claudia Venegas Cuzmar, Sharon Deem, Jacqueline M. Doyle

**Affiliations:** a Department of Biological Sciences, Towson University, Towson, MD, USA; b The Maryland Zoo in Baltimore, Baltimore, MD, USA; c Noel Kempff Mercado Natural History Museum, Santa Cruz de la Sierra, Bolivia; d Saint Louis Zoo, Saint Louis, MO, USA

**Keywords:** Genomics, conservation, endangered, cetacean, management

## Abstract

*Inia boliviensis*, the endemic Bolivian river dolphin, is threatened by anthropogenic activities including diversion of waterways for irrigation of agricultural fields, habitat degradation through deforestation, and the construction of hydroelectric dams. Within the department of Santa Cruz in Bolivia, conservation partners are committed to the capture and relocation of river dolphins that have been isolated through seasonally changing waterways, which are exacerbated by anthropogenic changes to local rivers’ courses. During these rescue attempts, tissue samples were taken to better understand the genetic composition of the fragmented populations. Herein, we describe the newly sequenced and assembled Bolivian river dolphin mitochondrial genome. The genome assembly is 16,591 base pairs in length, with an overall base composition of 32.75% adenine, 25.85% thymine, 28.35% cytosine, and 13.05% guanine. This resource will pave the way for a better understanding of Bolivian river dolphin population genetics, which will help inform effective management of these vulnerable populations.

## Introduction

*Inia boliviensis* (D’Orbigny 1834) is endemic to the waters of the upper Madeira River basin of Bolivia. These dolphins (locally referred to as *bufeos*) are morphologically distinct (Pilleri and Gihr 1977; Emin-Lima et al. 2022), genetically differentiated, and largely isolated from the Amazon River dolphin, or *boto* (*Inia geoffrensis*) (Ruiz-García et al. 2008; Hollatz et al. 2011; Hrbek et al. 2014; Gravena et al. 2014) by a series of rapids and the Teotȏnio waterfalls. However, the same anthropogenic pressures that affect *botos* are also harming the remaining populations of Bolivian river dolphins. Habitat degradation and fragmentation through building of hydroelectric dams, deforestation, and water diversion for agricultural irrigation have sharpened conservation concerns for these vulnerable populations (Aliaga-Rossel 2015). A team based at the Noel Kempff Mercado Natural History Museum in the department of Santa Cruz has committed to the relocation of geographically isolated individuals (Aliaga-Rossel and Escobar-WW 2020). These rescue attempts are completed in tandem with conservation partners and aim to mitigate harm from infrastructure and agricultural practices. In both 2018 and 2022, veterinarians from the Maryland Zoo in Baltimore and Saint Louis Zoo assisted in rescues of stranded *bufeos*, collecting individual biometric and medical data, and tissue samples for genetic analysis.

**Figure 1.**
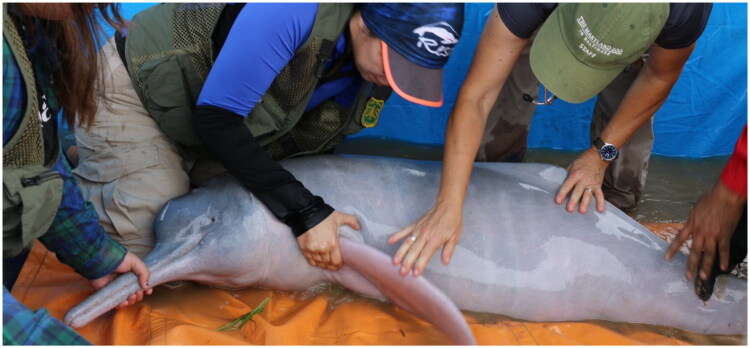
Pictured is an *Inia boliviensis* individual during a rescue. *Inia boliviensis* individuals are distinguished from *Inia geoffrensis* individuals by cranial differences including a smaller braincase and narrower, longer rostrum (Emin-Lima et al. 2022). Additionally, these species are geographically separated by rapids along the Madeira River. Photo credit: Rosario Arispe.

Compared to marine cetaceans, river dolphins at large are grossly underrepresented in genomic research. For example, publications on the genomics of Delphinidae species outnumber those available for *Iniidae* species eight-fold (Scopus searches 1 and 2) (SCOPUS search terms “genomics + Delphinidae”; SCOPUS search terms “genomics + Iniidae”). However, researchers have used mitochondrial markers and nuclear microsatellites to better understand the evolutionary history (Banguera-Hinestroza et al. 2002; Ruiz-García, 2010; Gravena et al. 2014, 2015), genetic richness (Ruiz-García et al. 2008, 2024; Aliaga-Rossel, 2015), gene flow (Vianna et al. 2010; Ruiz-García 2010; Gravena et al. 2014, 2015; Aliaga-Rossel 2015; Ruiz-García et al. 2024), and effective population sizes (Gravena et al. 2014) of remaining, fragmented populations of *Inia boliviensis*. Assembling the complete *bufeo* mitogenome will be an essential aid in documenting genetic variation among populations and between species (Figure 1).

## Materials and methods

18_3 was part of a group of dolphins that had become isolated in an oxbow lake (63°27’30”W and 16°12’20”S) over the course of many years, becoming completely isolated from the Rio Grande in Santa Cruz, Bolivia. In 2018, a team corralled the animal with a net system and moved it to land for assessment and transport. 18_3 was then placed in a padded, wet pool where it received a veterinary exam including blood samples, biometric data collection, and satellite tag placement. The tissue that was displaced during tag placement in the dorsal fin was stored in 90% ethanol for later analysis. The animal was transported and released ∼18 km from the capture site in adjacent waters of the Rio Grande. The tissue sample is stored at −20 °C at Towson University in Baltimore County, Maryland (Jacqueline Doyle, jdoyle@towson.edu) under voucher/accession number 18_3_S. We extracted DNA (deoxyribonucleic acid) using potassium acetate extraction (Sambrook and Russell 2000). A whole genome DNA library was prepared using a Kapa Hyper prep kit and sequenced with NovaSeq6000. We generated 594,598,924 paired-end, 150 bp reads. We used bbmap to subsample 10% of the first 200,000 paired-end reads in the raw sequence files before assembling the mitogenome using the MitoZ pipeline (Meng et al. 2019). The MitoZ pipeline incorporates fastp (Chen et al. 2018) for removing adaptors, discarding short reads and trimming poor quality bases from sequencing reads and MEGAHIT (Li et al. 2015) for genome assembly. MitoZ also incorporates a Perl-based script, MiTfi and infernal-1.1.1 (Nawrocki and Eddy 2013; Meng et al. 2019); to annotate the assembly. Read depths averaged 72 replicates and are represented in Supplementary Figure S1. Alignment was performed using ClustalW (Thompson et al. 1994) algorithm in Molecular Evolutionary Genetics Analysis (MEGA) software (Tamura et al. 2021). The Maximum Likelihood phylogenetic tree was constructed using complete mitogenome sequences in MEGA software (Tamura et al. 2021). We used the Tamura-Nei model to calculate distance and bootstrapping with 1000 replicates to evaluate the statistical support for the tree (Tamura and Nei 1993).

## Results

**Figure 2.**
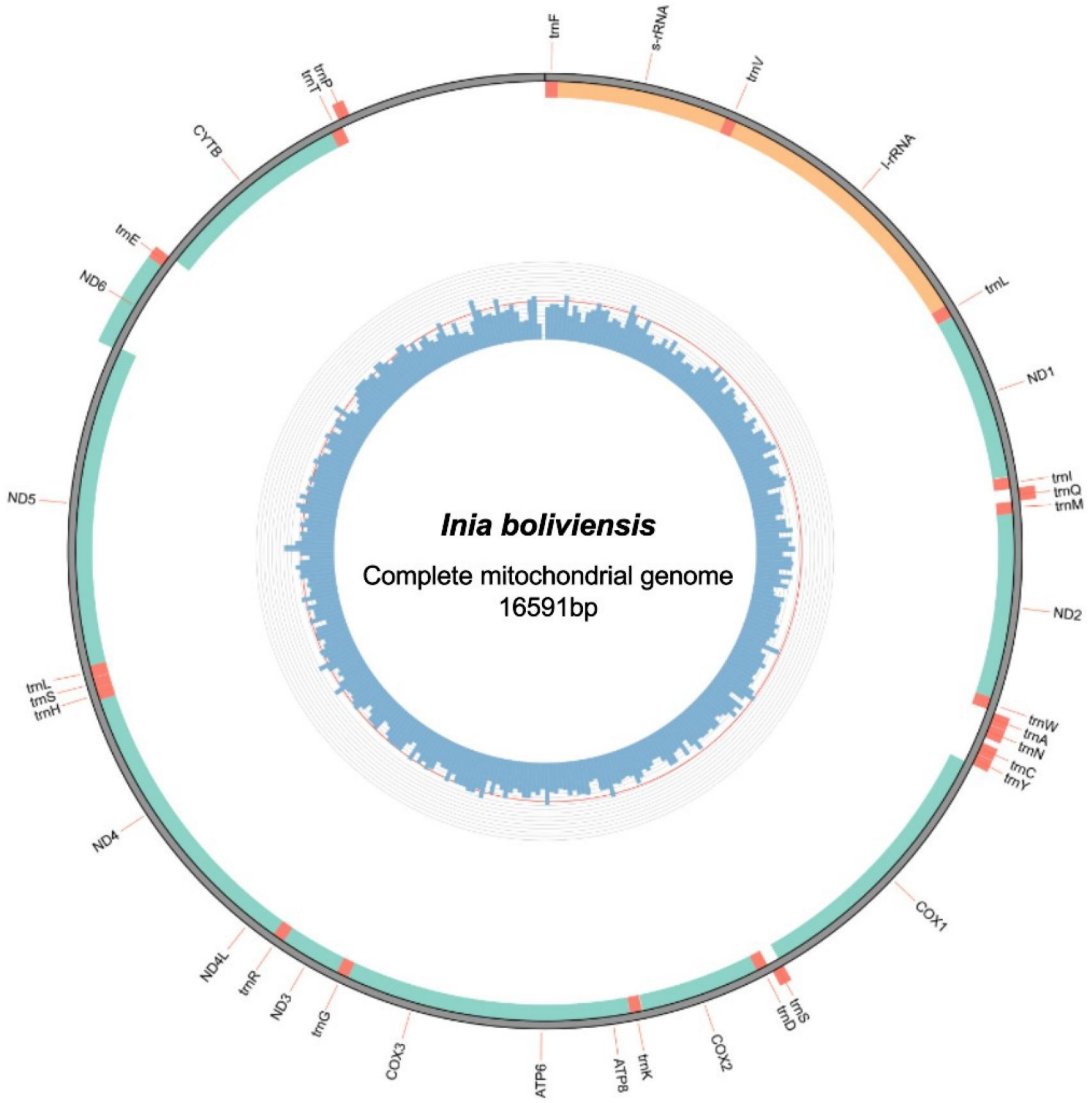
The complete, circular mitochondrial genome for *inia boliviensis*. Genes are color-coded with red representing tRNA genes, orange as rRNA genes, and green as protein coding genes (PCGs). Guanine, cytosine (GC) content is indicated by the blue ring within the genome. The red line indicates 50% GC content. This figure was generated using Circos (Krzywinski et al. 2009) as implemented by the MitoZ pipeline (Figure 3).

The mitogenome of *Inia boliviensis* is 16,591 base pairs in length, with an overall base composition of 32.75% adenine, 25.85% thymine, 28.35% cytosine, and 13.05% guanine. The mitogenome includes 13 protein-coding genes (PCGs), 22 transfer RNA genes (tRNAs), and 2 ribosomal RNA genes (rRNAs) (Figure 2). The major strand has 12 PCGs (*ND1, ND2, COX1, COX2, ATP8, APT6, COX3, ND3, ND4L, ND4, ND5,* and *CYTB*), 14 tRNA genes (*tmT^(ugu)^,tmF^(gaa)^, trnV^(uac)^, trnL^(uaa)^, trnl^(gau)^, trnm^(cau)^, trnW^(uca)^, trnD^(guc)^, trnK^(uuu)^, trnG^(ucc)^, trnR^(ucg)^, trnH^(gug)^, trnS^(gcu)^,* and *trnL^(uag)^)*, and both rRNA genes (*s-rRNA* and *l-rRNA).* The minor strand has one PCG (*ND6*) and eight tRNA genes (*tmP^(ugg)^, trnQ^(uug)^, trnA^(ugc)^, trnN^(guu)^, trnC^(gca)^, trnY^(gua)^, trnS^(uga)^,* and *trnE^(uuc)^)*.

**Figure 3.**
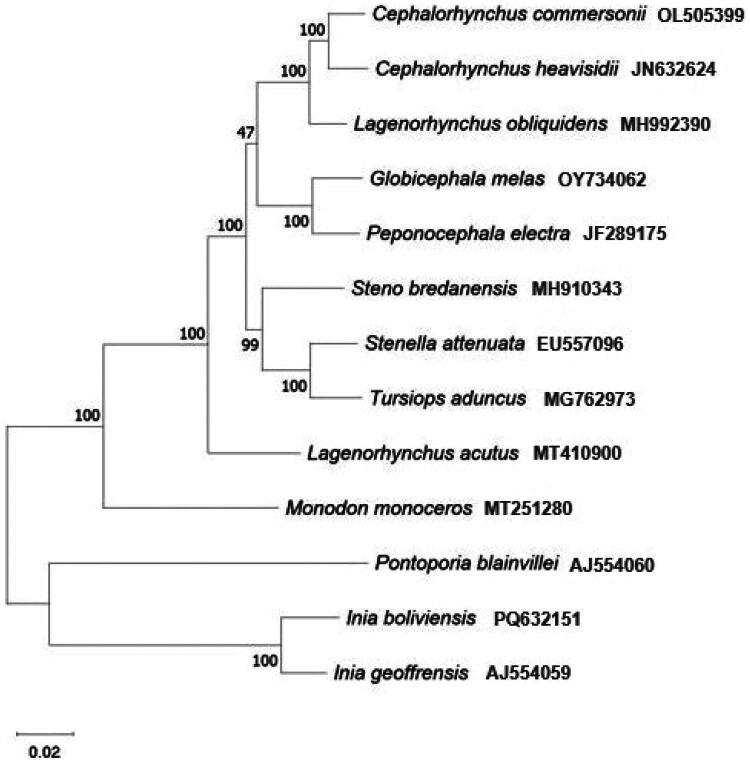
The maximum likelihood tree of 13 cetacean mitogenomes representing 80% or greater percent identity to 18_3 mitogenome in Standard Nucleotide BLAST search (National Center for Biotechnology Information [NCBI] Nucleotide Basic Local Alignment Search Tool [BLAST] 2024). The mitochondrial genomes of the following species were used: *Cephalorhynchus commersonii* OL505399 (Wang 2021)*, Cephalorhynchus heavisidii* JN632624 (Hassanin et al. 2012), *Lagenorhynchus obliquidens* MH992390 (Jackman et al. 2019), *Steno bredanensis* MH910343 (Park et al. 2019), *Stenella attenuata* EU557096 (Xiong et al. 2009), *Tursiops aduncus* MG762973 (Gray et al. 2018), *Globicephala melas* OY734062 (2003), *Peponocephala electra* JF289175 (Vilstrup et al. 2011), *Lagenorhynchus acutus* MT410900 (Margaryan 2020), *Monodon monoceros* MT251280 (Louis et al. 2020), *Pontoporia blainvillei* AJ554060 (Arnason et al. 2004), *Inia boliviensis* PQ632151 (Doyle et al. 2025), and *Inia geoffrensis* AJ554059 (Siciliano et al. 2016). Numbers at each node are bootstrap values and scale represents branch length.

## Discussion and conclusion

This mitogenome assembly will contribute to our understanding of evolutionary relationships in the *Inia* lineages. The taxonomy of *Inia* is of ongoing debate and arguments have been made for 1 to 4 distinct species (Hrbek et al. 2014; Siciliano et al. 2016; Society for Marine Mammalogy Committee on Taxonomy [SMMCT] 2021; Emin-Lima et al. 2022) Furthermore, the complete mitogenome will likely inform management strategies. For example, newly discovered mitochondrial markers, coupled with eDNA techniques, could be used to noninvasively test for the species presence in areas where it is not feasible to directly sample animals, or where species identification may be challenging (Ruiz-García et al. 2024). In addition, the annotation of mitochondrial markers will guide documentation of genetic variation and potentially inform the relocation of dolphins rescued from isolated waterways.

## Supplementary Material

Supplementary Materials.pdf

## Data Availability

The genome sequence data that support the findings of this study are openly available in GenBank of NCBI at [https://www.ncbi.nlm.nih.gov/] under accession no. PQ632151. The associated BioProject, SRA, and Bio-Sample numbers are PRJNA1226616, SRR32544792, and SAMN46945173, respectively.

## IUCN threatened or endangered species research statement

All research was performed in adherence with the IUCN Policy Statement on Research Involving Species at Risk of Extinction. Sample collection was non-lethal, responsible, and performed under the previously listed NMFS and CITES authorizations. Additionally, handling of river dolphins for tissue sample collection was secondary to the rescue and relocation efforts that made it possible for these isolated dolphins to once again access contiguous habitat and conspecifics.
