# Supplementary material for: Mitochondrial genome of a Bolivian River Dolphin (Inia boliviensis)
Source: Mitochondrial DNA B Resour. 2025 Aug 11;10(9):804–8. doi: 10.1080/23802359.2025.2544682 (PMC12340936; doi:10.1080/23802359.2025.2544682)
Supplement: Supplementary Materials.pdf [file TMDN_A_2544682_SM3935.pdf]

Mitochondrial genome of a Bolivian River Dolphin (*Inia boliviensis*)

Authors: Kristin Coury*[1], Ellen Bronson DVM[2], Claudia Venegas Cuzmar[3], Sharon Deem DVM, PhD[4], Jacqueline M. Doyle, PhD[1]

[1]*Department of Biological Sciences, Towson University, 8000 York Road, Towson, Maryland 21252, USA.*

[2]*The Maryland Zoo in Baltimore, 1 Safari Place, Baltimore, Maryland 21217, USA.*

[3]*Noel Kempff Mercado Natural History Museum, Av. Irala #565, Av. Ejercito Nacional y, Santa Cruz de la Sierra, Bolivia*

[4]*Saint Louis Zoo, 1 Government Drive, Saint Louis, Missouri 63110, USA.*

** = corresponding author*

KC: kcoury@towson.edu; ORCID number: 0009-1487-9072

Kristin Coury is a graduate (2023) of the Environmental Science Master's Program at Towson University in Maryland, USA.

EB: ellen.bronson@marylandzoo.org; ORCID number: 009-0002-8153-6579

Dr. Ellen Bronson, DVM is the Senior Director of Animal Health, Conservation, and Research at The Maryland Zoo in Maryland, USA.

CVC: cvenegascuzmar@gmail.com; ORCID number: 0009-0005-3916-2044

Claudia Venegas Cuzmar is a volunteer associate researcher at the Noel Kempff Mercado Natural History Museum and the coordinator of the Dolphin Conservation Program.

SD: deem@stlzoo.org; ORCID number: 0000-0002-2549-3636

Dr. Sharon Deem, DVM, PhD is the Director of the Saint Louis Zoo Institute for Conservation Medicine (ICM) at the Saint Louis Zoo in Missouri, USA.

JMD: jdoyle@towson.edu; ORCID number: 0000-0002-1626-0398

Dr. Jacqueline M Doyle, PhD is an associate professor at Towson University in Maryland, USA.

Supplementary Materials

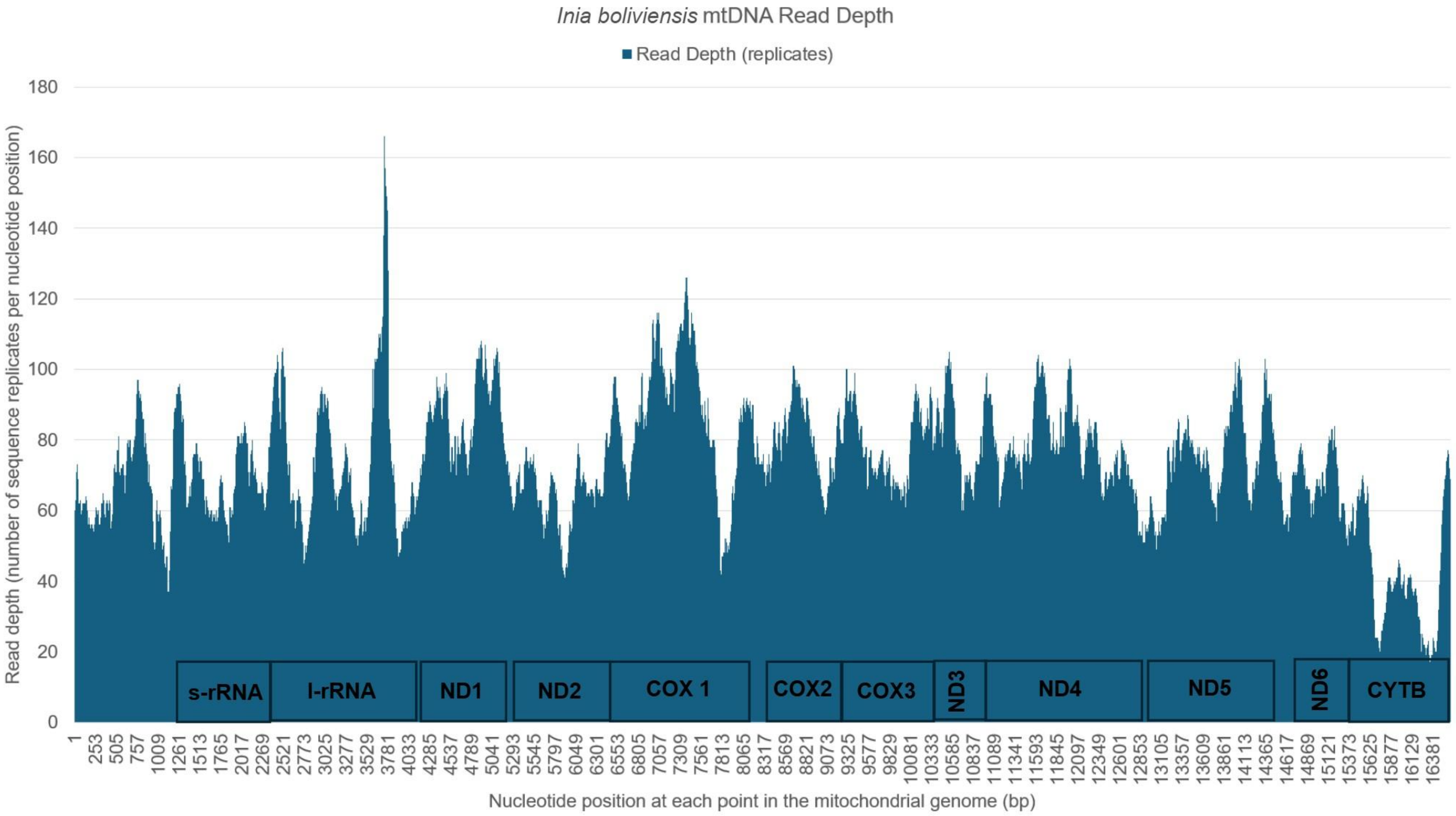

Supplementary Figure S1. Read depth, as measured by number of sequence replicates at each nucleotide position, of the *Inia boliviensis* 18_3 mitochondrial genome. BWA[1], as implemented in the MitoZ pipeline, was used to map sequence reads to the mitochondrial genome and calculate read depth. The figure was generated using Microsoft Excel. Notable genes are labeled above the x-axis.

Supplementary Materials Only References

1. Li, H., Durbin, R. Fast and accurate short read alignment with Burrows-Wheeler transform. *Bioinformatics* **25,** 1754-1760 (2009).
